# Prone Position Ventilation in Severe ARDS due to COVID-19: Comparison between Prolonged and Intermittent Strategies

**DOI:** 10.3390/jcm12103526

**Published:** 2023-05-17

**Authors:** George Karlis, Despina Markantonaki, Sotirios Kakavas, Dimitra Bakali, Georgia Katsagani, Theodora Katsarou, Christos Kyritsis, Vasiliki Karaouli, Paraskevi Athanasiou, Mary Daganou

**Affiliations:** 1 ICU, Thoracic Diseases General Hospital “Sotiria”, 115 27 Athens, Greece; despinamark@gmail.com (D.M.); demevbak@gmail.com (D.B.); gkatsagani@hotmail.com (G.K.); katsaroutheo@gmail.com (T.K.); silkarol@yahoo.gr (V.K.); athanasiou.par@hotmail.com (P.A.); mdaganou@hotmail.com (M.D.); 2 Henry Dunant Hospital Center, 115 26 Athens, Greece; sotikaka@yahoo.com

**Keywords:** ARDS, prone position, COVID-19, mechanical ventilation

## Abstract

Ventilation in a prone position (PP) for 12 to 16 h per day improves survival in ARDS. However, the optimal duration of the intervention is unknown. We performed a prospective observational study to compare the efficacy and safety of a prolonged PP protocol with conventional prone ventilation in COVID-19-associated ARDS. Prone position was undertaken if P/F < 150 with FiO_2_ > 0.6 and PEEP > 10 cm H_2_O. Oxygenation parameters and respiratory mechanics were recorded before the first PP cycle, at the end of the PP cycle and 4 h after supination. We included 63 consecutive intubated patients with a mean age of 63.5 years. Of them, 37 (58.7%) underwent prolonged prone position (PPP group) and 26 (41.3%) standard prone position (SPP group). The median cycle duration for the SPP group was 20 h and for the PPP group 46 h (*p* < 0.001). No significant differences in oxygenation, respiratory mechanics, number of PP cycles and rate of complications were observed between groups. The 28-day survival was 78.4% in the PPP group versus 65.4% in the SPP group (*p* = 0.253). Extending the duration of PP was as safe and efficacious as conventional PP, but did not confer any survival benefit in a cohort of patients with severe ARDS due to COVID-19.

## 1. Introduction

The COVID-19 pandemic created unprecedented pressure on healthcare systems worldwide and subsequently provoked significant changes in the organization of healthcare services. Coronavirus disease has a broad spectrum of clinical manifestations ranging from asymptomatic infection to critical illness, most frequently presenting as an acute hypoxemic respiratory failure that meets the Berlin definition of acute respiratory distress syndrome (ARDS). Patients with COVID-19-associated ARDS commonly require intensive care unit (ICU) admission and invasive mechanical ventilation and have high mortality rates [1,2]. Whether ARDS due to COVID-19 and ARDS due to other etiologies are similar has been a matter of debate. It seems that COVID-19 pneumonia is a specific disease with distinct features, namely the dissociation between the severity of the hypoxemia and the maintenance of relatively good respiratory mechanics, as well as the common (micro)thrombosis in the pulmonary vasculature [3].

It is well established that early application of prone-positioning (PP) sessions of at least 12 h improve survival in moderate-to-severe ARDS [4,5]. The beneficial effect of prone ventilation is likely attributed to better ventilation-perfusion matching, lung recruitment and protection from ventilator-induced lung injury (VILI) [6]. Current guidelines on the management of ARDS strongly recommend the use of PP for 12 to 16 h per day in patients with a P/F ratio ≤ 150 mmHg [4,5,7]. However, the optimal duration of the intervention to gain maximum benefit is not known. During COVID-19 pandemic prone ventilation was widely adopted as a prominent therapeutic intervention for patients receiving mechanical ventilation. Retrospective data from this patient population showed that early application of PP is associated with improved oxygenation and reduced hospital mortality [8,9]. 

One of the challenges of PP is that it can increase the workload for the ICU staff in a period of crisis. To overcome this problem, it was suggested to implement a prolonged pronation protocol, beyond the usual 16 h, aiming to reduce the number of pronation cycles per patient. Nevertheless, the intervention is not without risks. The most severe complications are accidental extubation, airway obstruction, central venous catheter or arterial catheter dislocation, pressure ulcers, peripheral nerve palsies and musculoskeletal injuries [10]. There are reports that prolonged prone ventilation is feasible and relatively safe [11,12], but comparison with standard PP has been scarce. 

We sought to examine the efficacy and safety of a prolonged PP protocol compared to the standard of care.

## 2. Materials and Methods

We conducted a prospective observational study. General Hospital of Thoracic Diseases “Sotiria” is a tertiary public hospital that serves as the main referral center for COVID-19 in Athens, Greece. The study was conducted in a 12-bed COVID-19 ICU during a 6-month period. Patient demographics, clinical and mechanical ventilation (MV) variables were entered into an electronic spreadsheet and cross-validated with source documentation in real-time. The study was approved by the local Institutional Review Board (protocol number 172/24-05-2021). The need for informed consent was waived.

Lung protective ventilation was universally applied to all study patients, namely a tidal volume of 6–8 mL/PBW with PEEP titration to achieve a driving pressure <14 cm H_2_O. The level of PEEP was applied and modified by the treating physician. PP was initiated for severe hypoxemia defined as P/F ratio < 150 mmHg with FiO_2_ > 0.6 and PEEP > 10 cm H_2_O. Patients with severe hemodynamic instability, pregnancy, recent cardiac or abdominal surgery, and unstable fractures were not candidates for prone ventilation in our study. In the prolonged PP (PPP) group patients were proned for more than 24 h whereas the standard PP (SPP) group included patients proned for 24 h or less. The cut-off of 24 h was based on a recent study of ARDS patients which suggested that it is beneficial to prolong PP sessions to 24 h and extend it further if the P/F ratio remains below 150 [13]. We left the decision for the duration of PP solely at the discretion of the treating physician, according to a guiding protocol stating that return to the supine position would be performed after at least 16 h if the P/F ratio was above 150 and if an experienced staff was available. For safety reasons, repositioning during the night shift was avoided, unless it was deemed necessary. Pronation cycles were stopped when the P/F ratio remained >150 mmHg in a supine position. Oxygenation parameters and respiratory mechanics were recorded for the first pronation cycle before PP, at the end of the cycle and 4 h after supine repositioning. We included all the intubated patients > 18 years old, with a positive PCR for SARS-CoV-2, who underwent at least one cycle of PP during the specified time period. Patients proned for less than 4 h were excluded from the analysis.

Prone positioning and repositioning to the supine position were performed manually by experienced ICU staff according to a standardized protocol. Normally, 5 healthcare professionals were involved with the pronation/supination of non-obese patients, while 7 or more were involved if the patient was obese or morbidly obese. Alternating pressure air mattresses were used in all patients. Foam wedges, foam dressings, gel rings and pillows were used for pressure injury prevention. Alternating arm and head repositioning in the “swimming position” were performed every 6–8 h. Sedation and analgesia were titrated to achieve deep sedation (Richmond Sedation Agitation Scale score of 4–5) and neuromuscular blockade was administered to all patients during PP. Pressure wounds and other complications were recorded daily by bedside nurses, until the end of the pronation cycles.

### 2.1. Outcomes

The primary clinical outcomes were changes in oxygenation and respiratory mechanics during and after PP and the number of PP cycles. The secondary outcome was 28-day survival. A subgroup analysis of obese patients (BMI > 30 kg/m^2^) was additionally performed. We also examined the safety and the complications of the procedure.

### 2.2. Statistical Analysis

Variables were first tested for normality using the Kolmogorov–Smirnov criterion. Quantitative variables were expressed as mean (Standard Deviation) or median (interquartile range). Qualitative variables were expressed as absolute and relative frequencies. Student’s *t*-tests and Mann–Whitney tests were used for the comparison of continuous variables between the two groups. For the comparison of proportions chi-square and Fisher’s exact tests were used. Pearson correlation coefficients (r) were used to explore the association of two continuous variables. Repeated measurements analysis of variance (ANOVA) was adopted to evaluate the changes observed in respiratory parameters over the follow-up period, between the two groups. Logistic regression analysis in a stepwise method (*p* for entry 0.05, *p* for removal 0.10) was used in order to find independent factors associated with 28-day survival. Adjusted odds ratios (OR) with 95% confidence intervals (95% CI) were computed from the results of the logistic regression analysis. All reported *p* values are two-tailed. Statistical significance was set at *p* < 0.05 and analyses were conducted using SPSS statistical software (version 26.0).

## 3. Results

From March 2021 to August 2021, we recorded 68 consecutive intubated patients with COVID-19-associated ARDS who underwent prone ventilation. Five patients were excluded because PP was terminated in less than 4 h due to hemodynamic instability or worsening of oxygenation. The final study sample consisted of 63 patients (63.5% males), with a mean age of 63.5 years. Thirty-seven patients (58.7%) underwent prolonged prone position (PPP group) and 26 (41.3%) standard prone position (SPP group). The median PP duration for the SPP group was 20 h (IQR: 20–22) and for the PPP group 46 h (IQR: 40–48), *p* < 0.001. The cumulative duration of pronation was longer for the PPP group. Patients’ characteristics by group are presented in Table 1. No significant differences were found between the two groups, except for a higher proportion of obese patients among the PPP group. All patients received corticosteroids while tocilizumab was administered to similar proportions in both groups.

The change in P/F ratio was similar across all time points between SPP and PPP groups, in the total sample and in the subgroup of obese patients. In both groups, the P/F ratio during and after PP was significantly higher compared to the baseline (Table 2).

**Figure 1:**
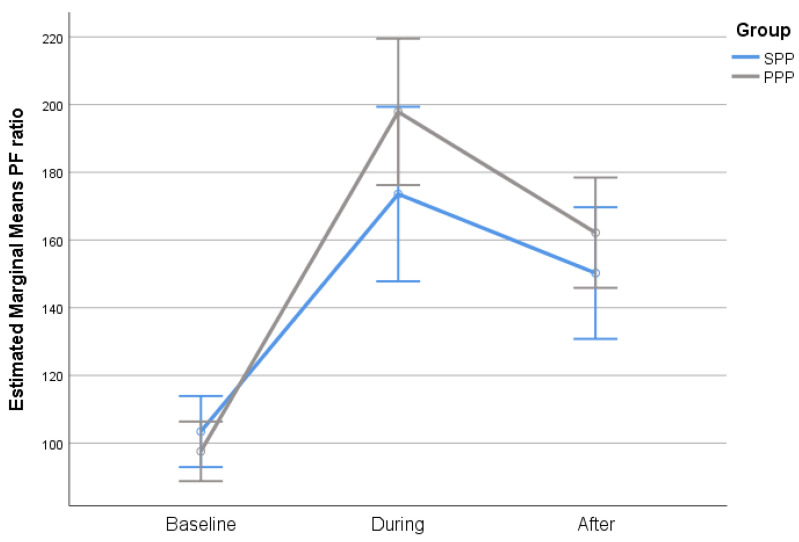
P/F ratio change, by group.

The degree of P/F ratio change from baseline to the end of the first PP cycle to 4 h after supination was similar in both groups (Figure 1). Furthermore, PP duration was not correlated with the P/F ratio (r = 0.18; *p* = 0.161). 

The change in respiratory parameters by the group throughout the follow-up period is presented in Table 3. No significant differences were found between SPP and PPP groups at any time point. Pplat was slightly lower during the maneuver compared to baseline in both groups, while after supination it remained lower than baseline only in the PPP group. However, because PEEP was also lower during and after the maneuver, DP and Cstat were similar throughout time. 

No significant difference was found in 28-day survival between the two groups. The number of pronation cycles was also comparable [median (IQR), 2(1–3) for SPP vs. 1(1–2) for PPP group]. Seven patients (26.9%) in the SPP group and 5 (13.5%) in the PPP group required 3 or more pronation cycles. No major complications were encountered in either group after the completion of the required pronation cycles. Facial edema and pressure injuries in stage I were recorded in six patients during PPP and in four patients during SPP, while one patient in each group developed a stage II facial pressure ulcer and one patient in the PPP group developed a stage III facial injury and periorbital edema (Table 4). Similar results were recorded for obese patients as shown in Table 5. 

After conducting multiple logistic regression, in a stepwise method, it was found that the number of PP cycles, APACHE II and PaCO_2_ at baseline (before pronation) were independently associated with 28-day survival. Specifically, a higher number of cycles, higher APACHE II score and higher PaCO_2_ at baseline were significantly associated with a lower probability of surviving (Table 6). 

## 4. Discussion

In a cohort of COVID-19 intubated patients with severe ARDS, in whom protective ventilation was applied, a prolonged prone positioning protocol was not shown to confer any advantage in improving oxygenation and respiratory mechanics compared to the traditional strategy of daily prone ventilation. Furthermore, it was not associated with significantly fewer total pronation cycles. Twenty-eight-day survival was similar between the two groups. The intervention was feasible and safe with only minor observed complications.

Prone position ventilation for at least 16 h has been shown to reduce mortality in patients with ARDS and a P/F ratio of <150 mmHg. This beneficial effect does not depend on gas exchange improvement but is rather attributed to protection from VILI by reducing overdistension of non-dependent and enhancing alveolar recruitment of dependent lung zones, leading to more homogeneous lung expansion and reducing lung stress and strain [4]. Prone ventilation was widely adopted during the COVID-19 pandemic. While the LUNG SAFE study in 2016 [14] reported the application of prone positioning in only 16.3% of patients with moderate to severe ARDS, the intervention was used in more than 70% of mechanically ventilated patients with COVID-19 [15,16].

Early prone ventilation has been associated with improved survival among COVID-19 patients [9]. However, it is a labor-intensive procedure requiring at least 5 highly trained ICU professionals to execute each pronation and supination maneuver [17]. In conditions of increased workload, as was the case during the pandemic, prolongation of the duration of prone ventilation to more than 24 h seemed an attractive option to reduce the burden of this life-saving intervention. Furthermore, prolonging PP has a physiological rationale as there is data suggesting that the beneficial effects of PP may persist for at least up to 24 h for some patients [13], while supination is often accompanied by de-recruitment events. Prior studies in COVID-19 patients with ARDS showed that prolonged PP is efficacious and safe when performed by experienced staff [12]. However, the efficacy of the maneuver compared to the standard practice of shorter duration daily cycles has not been extensively studied. In a single-center study of patients with pneumonia and ARDS, Jochmans et al. reported that the maximum physiological beneficial effect of PP was obtained between 16 and 19 h in most patients and extending pronation for more than 24 h offered no survival benefit [13]. In patients with COVID-19-related ARDS, improvement of oxygenation with proning has been associated with lower mortality [18]. 

In a recent retrospective study of intubated COVID-19 patients, Okin et al. [19] reported reduced mortality and fewer pronation-supination cycles for the prolonged PP compared to the standard PP. The authors found no difference in oxygenation improvement between PPP and SPP, measured as the change in P/F ratio within 6 h of pronation. In the present study, we found a similar increase in the P/F ratio between prolonged and intermittent proning during the maneuver and up to 4 h after supination. Furthermore, there was no correlation between PP duration and the P/F ratio. It seems that extending proning beyond 24 h confers little further improvement in oxygenation. Changes in respiratory mechanics were of little clinical significance in both groups. Okin et al., attribute the beneficial effect on survival to the reduced de-recruitment events associated with fewer supination sessions. Despite similar patient characteristics and similar duration of prolonged pronation protocol between the two studies, we did not find a survival advantage of PPP over SPP. However, there are caveats that should be addressed. First, 28-day mortality rates in our cohort are similar to those in Okin’s study (21.6% vs. 25.5% for the PPP group and 34,6% vs. 34.9% for the SPP group). Therefore, the lack of significance is probably due to the smaller sample size of our study. Second, although we found no difference in the total number of performed PP cycles between groups, the number of cycles was an independent risk factor for mortality. The more the pronation cycles, the greater the mortality, lending support to the assumption that de-recruitment associated with repeated supination may be injurious to the lung, may worsen VILI and may contribute to mortality. It can be speculated that PPP may reduce mortality when it results in fewer pronation and supination events. Third, in our study, a higher proportion of obese patients were included in PPP than in the SPP group. Since the decision for the duration of PP was solely at the discretion of the treating physicians, we can only assume that obese patients were deemed more appropriate candidates for prolonged pronation to reduce the risk of complications and adverse events from frequent maneuvers. Several meta-analyses have shown that obesity is associated with increased severity and higher mortality among COVID-19 patients [20]. In a previous study, De Jong et al. reported a better response in oxygenation with prone ventilation among morbidly obese compared to non-obese patients with ARDS [21]. However, a subgroup analysis of our cohort showed that obese patients had no additional benefit with PPP compared to SPP.

The number of cycles per se was found to be associated with 28-day survival in our study. It is fair to assume that the sickest ARDS patients require more PP cycles while aiming to improve their oxygenation. Most of the patients in our cohort required 1 or 2 cycles which were akin to the corresponding study of Okin et al. [19] with a median of 2 cycles overall. Moreover, in a non-COVID population with ARDS due to pneumonia [13] a mean of 2.2 cycles per patient was reported, while in the PROSEVA trial which included ARDS from various etiologies, patients required a mean of 4 cycles [4]. It is not clear if there is an association between the etiology of ARDS and the response to PP. 

The rate of complications in our study was very low in both groups and almost exclusively consisted of minor facial pressure injuries. Pressure injuries are the most frequent complication of prone ventilation. In a retrospective study of 81 patients with COVID-19 who were ventilated in PP for a median of 39 h, 26% developed pressure injuries stage II and 2.5% stage III and IV. The cumulative duration of PP sessions, but not the duration of each session, was associated with the occurrence of pressure injuries [22]. Okin et al., reported an incidence of pressure sores of about 30%, with no difference between prolonged and intermittent PP. In our study, the prophylactic use of foam pads, foam dressings, gel rings and regular head repositioning, probably contributed to the low incidence of complications, which was also evident in the group of prolonged pronation.

There are several limitations to our study. It was a single-center non-randomized study with a potential risk for selection bias. The sample size was small and the study itself was not powered to detect a mortality difference. We recorded only immediate complications. There was no long-term follow-up and therefore we might have missed late complications such as plexopathy and nerve damage [23]. Moreover, the use of prophylactic measures along with the accumulated experience of our staff, possibly resulted in a low rate of early complications, which might not be generalizable to all ICU settings.

However, the present study confirms the feasibility and safety of prolonged prone ventilation among patients with severe ARDS caused by COVID-19 and highlights the urgent need for multi-center randomized trials comparing the efficacy of this maneuver with the standard technique of daily proning and supination in ARDS patients of various etiology. Should this approach prove to further improve mortality, it could be safely added to lung protective ventilation, which is so far the only lifesaving intervention in this patient population. In any case, this strategy seems to be a useful option in periods of increased ICU workload or for specific groups of patients, such as the obese.

## 5. Conclusions

Among intubated COVID-19 patients with severe ARDS, prolonging PP to more than 24 h was as safe and efficient as traditional PP, but it was not associated with survival benefits. 

## Figures and Tables

**Table 1:** Patients’ characteristics.

	Group		
	SPP *n* = 26	PPP *n* = 37	*p*
Gender, *n* (%)			
Males	15 (57.7)	25 (67.6)	0.42
Females	11 (42.3)	12 (32.4)	
Age, mean (SD)	66.5 (9.7)	61.5 (15.1)	0.14
BMI, mean (SD)	30.5 (5.5)	33.4 (7.0)	0.08
BMI			
Normal (18.5–24.9 kg/m^2^)	1 (3.8)	0 (0)	0.049
Overweight (25–29.9 kg/m^2^)	15 (57.7)	12 (33.3)	
Obese (>30 kg/m^2^)	10 (38.5)	24 (66.7)	
APACHE II, mean (SD)	19.3 (3.8)	19.5 (7.1)	0.89
Tocilizumab, *n* (%)	17 (65%)	21 (57%)	0.49
Vt (ml), median (IQR)	425 (375–450)	425 (375–475)	0.59
Vt (ml/PBW), median (IQR)	6.3 (6.2–6.7)	6.3 (6.2–6.7)	0.78
RR, median (IQR)	27.5 (25–32)	30 (27–32)	0.24
Time to proning, h, median (IQR)	22.5 (16–48)	20 (10–48)	0.59
Duration of 1st PP cycle, h, median (IQR)	20 (20–22)	46 (40–48)	<0.001
Cumulative duration of proning, h, mean (SD)	42.42 (22.27)	70.22 (38.29)	0.001

**Table 2:** P/F ratio in total sample and in obese patients.

Group		P/F Ratio (mmHg)			*p* ^2^			
		Baseline	During					
		Mean (SD)	Mean (SD)	Mean (SD)	During vs. Baseline	After vs. During	After vs. Baseline	*p* ^3^
Total sample	SPP	103.4 (25.8)	173.6 (59)	150.2 (32)	<0.001	0.09	<0.001	0.13
	PPP	97.6 (27.4)	197.9 (70.1)	162.2 (58.8)	<0.001	<0.001	<0.001	
	*p* ^1^	0.39	0.15	0.35				
Obese	SPP	105.1 (29.2)	176.5 (67.9)	152.8 (25.2)	0.002	0.69	0.004	0.43
	PPP	95.8 (27.1)	194.6 (67.8)	156.7 (48.7)	<0.001	0.014	<0.001	
	*p* ^1^	0.38	0.48	0.81				

^1^ *p*-value for group effect ^2^ *p*-value for time effect after Bonferroni correction ^3^ *p*-value from repeated measures ANOVA, regarding time × group effect.

**Table 3:** Changes of respiratory parameters.

		Baseline	During	After		*p* ^2^		
	Group	Mean (SD)	Mean (SD)	Mean (SD)	During vs. Baseline	After vs. During	After vs. Baseline	*p* ^3^
PaCO_2_ (mmHg)	SPP	50.9 (8.8)	47.8 (7.5)	48.3 (6.3)	0.22	>0.99	0.46	0.95
	PPP	50.8 (10.6)	47.6 (7.1)	48.5 (7.9)	0.08	>0.99	0.39	
	*p* ^1^	0.97	0.89	0.92				
PEEP (cm H_2_O)	SPP	12 (2.7)	11.1 (2.8)	11.2 (2.6)	0.012	>0.99	0.02	0.49
	PPP	12.3 (2.4)	11.6 (2.6)	11.2 (2.2)	0.018	0.29	<0.001	
	*p* ^1^	0.64	0.49	0.99				
Pplat (cm H_2_O)	SPP	25.3 (3.6)	23.6 (3.2)	23.9 (3.3)	0.009	>0.99	0.09	0.65
	PPP	25.5 (3.4)	23.9 (3.5)	23.5 (3.3)	0.003	>0.99	0.002	
	*p* ^1^	0.84	0.77	0.68				
DP (cm H_2_O)	SPP	13.4 (3.9)	12.2 (2.4)	12.2 (2.8)	0.16	>0.99	0.27	0.82
	PPP	12.9 (3.2)	12.1 (2.4)	12.2 (2.6)	0.25	>0.99	0.50	
	*p* ^1^	0.62	0.82	0.92				
Cstat (mL/cm H_2_O)	SPP	35.8 (10.2)	36.9 (11.6)	36.8 (10.7)	>0.99	>0.99	>0.99	0.75
	PPP	35.2 (9.5)	37.6 (9.3)	36.4 (8.8)	0.22	0.84	>0.99	
	*p* ^1^	0.81	0.77	0.87				

^1^ *p*-value for group effect ^2^ *p*-value for time effect after Bonferroni correction ^3^ *p*-value from repeated measures ANOVA, regarding time × group effect.

**Table 4:** Outcomes and complications.

	Group		
	SPP *n* = 26	PPP *n* = 37	*p*
28-day survival, *n* (%)			0.25
No	9 (34.6)	8 (21.6)	
Yes	17 (65.4)	29 (78.4)	
Number of cycles, median (IQR)	2 (1–3)	1 (1–2)	0.12
Complications, *n* (%)			>0.99
No	21 (80.8)	29 (78.4)	
Yes	5 (19.2)	8 (21.6)	

**Table 5:** Outcomes among obese patients.

		Group		
		SPP (*n* = 10)	PPP (*n* = 24)	
		Median	Median	*p*
28-day survival	No	4 (40)	5 (20.8)	0.39
	Yes	6 (60)	19 (79.2)	
Number of cycles, median (IQR)		2 (2–3)	1 (1–2)	0.19

**Table 6:** Logistic regression for 28-day survival.

	OR (95% CI)	*p*
Number of cycles	0.27 (0.12–0.63)	0.002
APACHE II	0.78 (0.66–0.92)	0.003
PaCO_2_ baseline (mmHg)	0.92 (0.86–0.99)	0.031

## Data Availability

The data presented in this study are available on request from the corresponding author. The data are not publicly available due to privacy restrictions.
